# Proteome Analysis of Human Follicular Thyroid Cancer Cells Exposed to the Random Positioning Machine

**DOI:** 10.3390/ijms18030546

**Published:** 2017-03-03

**Authors:** Johann Bauer, Sascha Kopp, Elisabeth Maria Schlagberger, Jirka Grosse, Jayashree Sahana, Stefan Riwaldt, Markus Wehland, Ronald Luetzenberg, Manfred Infanger, Daniela Grimm

**Affiliations:** 1 Max-Planck-Institute for Biochemistry, Scientific Information Services, 82152 Martinsried, Germany; schlagberger@biochem.mpg.de; 2 Clinic and Policlinic for Plastic, Aesthetic and Hand Surgery, Otto-von-Guericke-University, 39120 Magdeburg, Germany; sascha.kopp@med.ovgu.de (S.K.); markus.wehland@med.ovgu.de (M.W.); ronald.luetzenberg@med.ovgu.de (R.L.); manfred.infanger@med.ovgu.de (M.I.); dgg@biomed.au.dk (D.G.); 3 Department of Nuclear Medicine, University Hospital, University of Regensburg, 95053 Regensburg, Germany; jirka.grosse@klinik.uni-regensburg.de; 4 Department of Biomedicine, Aarhus University, 8000 Aarhus C, Denmark; jaysaha@biomed.au.dk (J.S.); sr@biomed.au.dk (S.R.)

**Keywords:** cellular compartments, mass spectrometry, proteomics, pathway analysis, random positioning machine

## Abstract

Several years ago, we detected the formation of multicellular spheroids in experiments with human thyroid cancer cells cultured on the Random Positioning Machine (RPM), a ground-based model to simulate microgravity by continuously changing the orientation of samples. Since then, we have studied cellular mechanisms triggering the cells to leave a monolayer and aggregate to spheroids. Our work focused on spheroid-related changes in gene expression patterns, in protein concentrations, and in factors secreted to the culture supernatant during the period when growth is altered. We detected that factors inducing angiogenesis, the composition of integrins, the density of the cell monolayer exposed to microgravity, the enhanced production of caveolin-1, and the nuclear factor kappa B p65 could play a role during spheroid formation in thyroid cancer cells. In this study, we performed a deep proteome analysis on FTC-133 thyroid cancer cells cultured under conditions designed to encourage or discourage spheroid formation. The experiments revealed more than 5900 proteins. Their evaluation confirmed and explained the observations mentioned above. In addition, we learned that FTC-133 cells growing in monolayers or in spheroids after RPM-exposure incorporate vinculin, paxillin, focal adhesion kinase 1, and adenine diphosphate (ADP)-ribosylation factor 6 in different ways into the focal adhesion complex.

## 1. Introduction

Multicellular spheroids (MCS) are interesting models of cancer [1,2]. They resemble natural tumors more than cell monolayers, but they are not as complex as those [3]. Spheroids may be generated in various ways [1]. One example is their exposure to real (r-µ*g*) and simulated microgravity (s-µ*g*) [3]. s-µ*g* can be achieved by application of the Random Positioning Machine (RPM), a device created to simulate microgravity on Earth. During RPM-exposure, human cells are oriented randomly with respect to the gravity vector, so that cell sedimentation does not occur [3]. In addition, the RPM is an interesting tool for novel applications, such as three-dimensional cell culturing as well as tissue engineering [3]. 

Technical characteristics for microgravity-dependent spheroid formation include the following: matrices are not required, frictional forces are extremely low, and cell-cell contact is established by cell surface features. Spheroid formation under microgravity in vitro includes a cell leaving a two-dimensional monolayer and joining neighbor cells in a three-dimensional manner. This process is supposed to mirror, in part, the change of a cancer cell’s kind of growth in vivo as it is observed during metastasis [4]. Hence, knowing the detailed cellular changes of spheroid formation may provide information about cellular events occurring during metastasis.

For more than a decade we have aimed to define the cellular mechanisms causing the transition from two- to three-dimensional growth in cells cultured under r-µ*g* or s-µ*g*. Meanwhile, we know that subconfluent cultures of FTC-133 thyroid cancer cells divide into two populations when they are exposed to r-µ*g* or s-µ*g* [5,6]. One continues to grow adherently on the bottom of the culture dish and the other one forms three-dimensional aggregates (spheroids), which detach from the surface of the culture dish and float in the culture medium. Interestingly, FTC-133 cells do not form spheroids when they are exposed to s-µ*g* created by an RPM or r-µ*g* during spaceflight after previously forming confluent monolayers [7]. Under these conditions, all cells continue to grow within a monolayer, independent of the gravitational force they are exposed to. 

In order to gain more information about the molecular mechanisms causing this differential growth behavior, we studied FTC-133 cells after encouraging or discouraging spheroid formation in vitro [5,6,7]. The harvested samples were analyzed by applying microscopy, gene array technology, quantitative real time-PCR, and Multi-Analyte Profiling with the aim to examine cell morphologies, gene expression patterns, and proteins which could be associated with the transition from a two- to a three-dimensional growth pattern [5,6,8,9]. Taken together, these efforts revealed that exposure of FTC-133 cells to microgravity enhances apoptosis and promotes nuclear factor kappa B (NFκB) p65 activities, while the caveolin 1 (*CAV-1*) gene is down-regulated during spheroid formation [6,10,11,12]. Most interestingly, several factors triggering angiogenesis were found in supernatants of thyroid cells exposed to the RPM [8]. In addition, Western blotting and mass spectrometry (MS) experiments revealed increased concentrations of several proteins, including vinculin, during growth on the RPM, while caveolin-1 proteins were enriched in confluent FTC-133 cultures which do not form spheroids, even if incubated on a RPM [7,12].

In this study, we applied advanced MS [13] and analyzed cells harvested after growth under five distinct conditions, as shown in Table 1. The proteins detected were assigned to their original cell compartments and the canonical pathways, wherein they are active, were assigned using modern computer programs. Thereby, we recognized that different proteins regulating the structure of the focal adhesion complexes were detectable in spheroid forming cells compared to cells that continued an adherent growth under equal conditions. The results challenged the conclusion that variable structures of the cell adhesion complex determine whether the cell leaves or perseveres in a given cell monolayer.

## 2. Results and Discussion

### 2.1. Spheroid Formation

In the present study, three flasks completely filled with cell suspension were incubated under each of the different culture conditions shown in Table 1. At the time of harvest, cells grown according to culture conditions 1, 4, 5, and 6 had formed monolayers only, while cells incubated under culture conditions 2 and 3 were separated into two parts: one grew as spheroids; the other one within adherent monolayers (Table 1). Hence, by these in vitro experiments, we reproduced earlier studies where FTC-133 thyroid cancer cells formed monolayers in plastic flasks if incubated under normal gravity. When subconfluent monolayers of this cell type were exposed to a spaceflight or to an RPM, one part of their cells formed spheroids [5,6]. Interestingly, spheroid formation did not occur when confluent monolayers were grown on the RPM or in space [7]. After harvest, the cells of three flasks of the same condition were pooled. This way, we obtained five samples (Table 1) usable for mass spectrometry (MS). Sample 1 contained cells grown in a monolayer, cultured under culture condition 1. Sample 2 included cells that formed spheroids under culture conditions 2 and 3, sample 3 was comprised of cells grown adherently under culture conditions 2 and 3. Sample 4 contained cells cultured under culture condition 4 and grown in a monolayer, as well as sample 5, which comprised cells grown as a monolayer only, although the cells had been cultured on the RPM (see culture conditions 5 and 6). As no spheroids were found when confluent monolayers were exposed to the RPM, a sample 6 could not be collected. All the samples were flash frozen immediately after harvest and stored until their preparation for MS.

### 2.2. Quantitative Overview on Proteins Detected by Mass Spectrometry

In order to see possible differences in the protein expression of the FTC-133 cells cultured under different conditions, MS was performed on the cell preparations of each of the five samples indicated above. The analyses revealed a total of 5924 different human proteins in FTC-133 cells. Of those, 3841 proteins were detected in all cells, independent of their incubation history. A total of 4419, 4505, 4544, 4621, and 4961 proteins were found in samples 1, 2, 3, 4, and 5, respectively (Table 1). Hence, applying modern electrospray ionization (ESI) MS technology we found much more than the 821 different proteins which we had detected about eight years ago when we applied matrix-assisted laser desorption/ionization (MALDI) technology after protein pre-separation with the help of electrophoretic techniques [14,15]. Interestingly, we confirmed more than 95% of the proteins, which had been detected previously in the examined thyroid cell lines FTC-133, ML-1, CGTH-W1, and HTU-5. Of the 35 proteins detected previously but not in the current experiment, 22 were present in ML-1, HTU-5, or CTGH-W1 cells, but only 15 were detectable in FTC-133 eight years ago. The first result might be explained by differences in the protein patterns in different thyroid cell lines. The second result could be due to a shift of isoforms as carbonic anhydrase 12, secernin 1, and high mobility group protein 1-like 10 appeared now instead of carbonic anhydrase 13, secernin 2, and high mobility group protein 1-like 1. However, it could also be due to the pre-separation procedure performed earlier as, for example, most of these proteins had either a rather high or low isoelectric point [16].

In order to gain information about the biological roles of the proteins detected by MS, we assigned the detected FTC-133 proteins to their original cellular compartments using Elsevier Pathway Studio® software. The analysis revealed that the coverage of major cellular compartments by the detected proteins was very similar in all cells treated by the different incubation methods. For example, 100% of all proteins typical for large ribosomal subunits and 93% of proteins characteristic for small ribosomal proteins were found in all five samples, while on average 27.6%, 30.2%, and 29.6% of known human membrane, cytoplasmic, and nuclear proteins, respectively, were detected (Table 2). Furthermore, less than 21% of proteins normally secreted into the extracellular space were detected in each sample. This may be explained by the washing of the cell samples prior to protein analysis. However, proteins of other cellular components such as mitochondria, endoplasmic reticulum, and nucleolus were found at average rates of 48.8%, 36.8%, and 49.8%, respectively, and of the focal adhesion complexes at 65% (Table 2). Comparing the numbers of Table 1 and Table 2, one may suggest that MS revealed around 40% of the proteins that can be found in the human cell proteome [13]. Of course, a protein not detected in our study does not directly imply that the protein is not expressed in FTC-133 cells.

### 2.3. Coverage of Different Canonical Pathways by the Proteins Detected in Different Cell Samples

**Figure 1:**
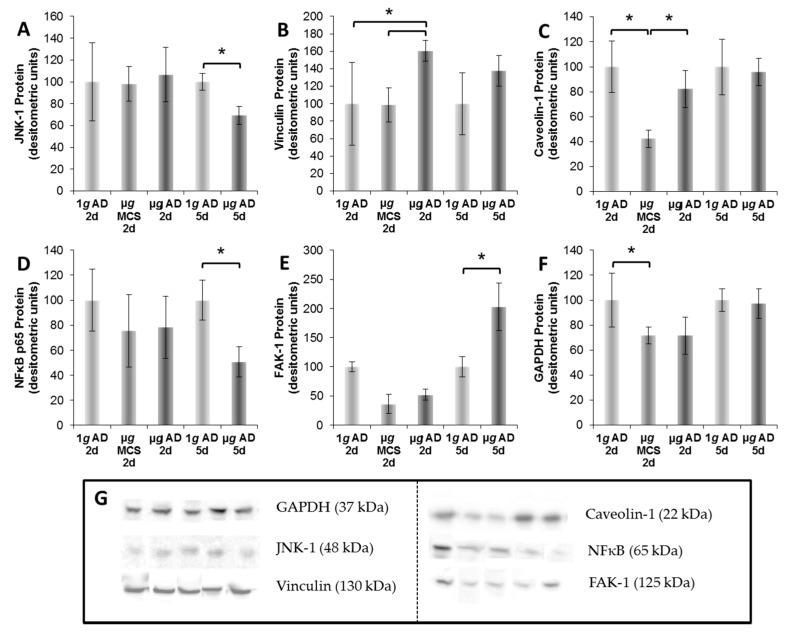
Western blot analyses and densitometric evaluation of FTC-133 cells exposed to culture conditions 1–5, normalized to the total protein content: (**A**) The c-Jun N-terminal kinase 1 (JNK-1) protein was not significantly altered when subconfluent monolayers were exposed to the Random Positioning Machine (RPM) for 3 days (d). JNK-1 was significantly reduced in adherent cells (AD) from culture condition 5 compared to that of the 1*g*-condition; (**B**) Vinculin was reduced in cells of multicellular spheroids (MCS) compared to that of AD (culture condition 3), and in AD 1*g* compared to that in AD (culture condition 3); (**C**) Caveolin 1 (Cav-1) protein was significantly reduced in MCS compared to that with 1*g* and AD (RPM); (**D**) Nuclear factor kappa B (NFκB) p65 protein was significantly decreased in the AD-RPM samples compared to that of the corresponding 1*g*-condition (culture condition 4); (**E**) Focal adhesion kinase 1 (FAK-1 also known as protein tyrosine kinase 2 or PTK2) protein was elevated in the AD of culture condition 5 compared to that in 1*g*; (**F**) Glycerinaldehyd-3-phosphat-Dehydrogenase (GAPDH) densitometric evaluation, normalized to the total protein content (Ponceau S red stain); (**G**) Western blot bands. * *p* < 0.05; *n* = 5 samples per group.

In further experiments, we sought information about the signaling behavior of the detected proteins by applying Ingenuity Pathway Analysis (IPA). This in silico method revealed 478 canonical pathways, in which the detected proteins covered at least 20% of the pathways’ proteins. A total of 229 of the indicated pathways comprised more than 20 contributing proteins. First, we took a closer look at pathways including caveolin 1 (CAV-1), which according to earlier results appears to inhibit spheroid formation [3,7]. This protein was detected by Western blotting (Figure 1). In MS measurement, it showed Label-free Quantitation (LfQ) values of 6.47 × 10^9^, 5.35 × 10^9^, 9.25 × 10^9^, 12.57 × 10^9^, and 12.86 × 10^9^ in samples 1, 2, 3, 4, and 5, respectively. The numbers clearly indicated that the spheroid forming cells (sample 2) produced less CAV-1 proteins than the controls (sample 1), while in samples 3–5 the cells produced more CAV-1. This result corresponds to the gene analyses described in [6,11]. It was partially confirmed by Western blot analysis shown in Figure 1C.

CAV-1 was found as a member of the following pathways: caveolar-mediated endocytosis signaling, endothelial nitric oxide synthase (eNOS) signaling, G beta gamma signaling, gap junction signaling, Gαi signaling, integrin signaling, nitric oxide signaling in the cardiovascular system, platelet-derived growth factor (PDGF) signaling, and virus entry via endocytosis. It is not known via which one of these pathways CAV-1 exerts its inhibition of spheroid formation. Therefore, based on earlier studies [17,18], we examined the proteins detected in samples 2 and 3 that are known to be members of the integrin signaling pathway first.

**Figure 2:**
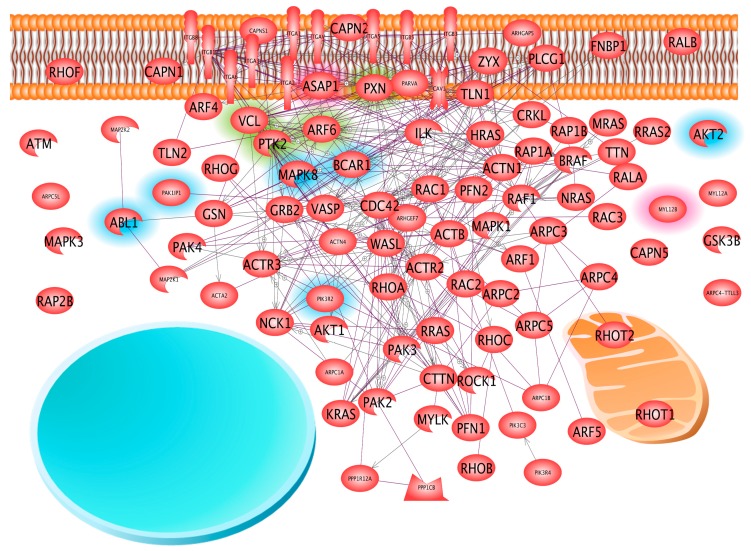
The interaction and localization of proteins belonging to the integrin signaling pathway and found in samples 2 and/or 3. The focal adhesion proteins paxillin (PXN), vinculin (VCL), PTK2 as well as ADB-ribosylation factor 6 (ARF6), marked by a green rim, may be influenced by the protein of ASAP1 (Arf-GAP with SH3 domain, ANK repeat and PH domain-containing protein 1), marked by a red rim, found only in sample 2 as well as by p130cas and Mitogen-activated protein kinase 8 (MAPK8) proteins, marked by a blue rim, found only in sample 3. Arrows indicate interaction; schemes of a membrane bilayer, a nucleus (circle) and a mitochondrion outline localization.

According to the IPA tool, the integrin signaling pathway comprises 207 proteins. We detected 98 and 102 of them in cells which grew in spheroids (sample 2) or monolayers (sample 3), respectively, although they had been subjected to equal conditions (see Table 1: culture condition 2 and 3) within the same flask. Amongst them were a considerable number of various types of membrane proteins including integrins, which had not been removed from the cells during the various wash steps. To our surprise, nuclear proteins linked to the integrin signaling pathways were not detected (Figure 2). Studying the 98 and 102 proteins mentioned above in detail, we found that 96 of the proteins detected in samples 2 and 3 and belonging to the integrin signaling pathway were identical. Only two of the 98 proteins of sample 2 and six of the 102 proteins of sample 3 were unique (Figure 2).

One of the proteins of the integrin pathway found only in sample 2 was the ASAP1 protein (Arf-GAP with SH3 domain, ANK repeat and PH domain-containing protein 1). As we demonstrated by Elsevier Pathway Studio^®^ analysis, this protein interacts with several other proteins (Figure 2). Amongst them are vinculin (VCL), a rather abundant protein detectable by MS with an average LfQ of 11.82 × 10^9^, and less abundant proteins such as paxillin (PXN) with an average LfQ value of 0.46 × 10^9^, focal adhesion kinase 1 (PTK2, average LfQ = 0.72 × 10^9^) as well as ADP-ribosylation factor 6 (ARF6, average LfQ = 0.6 × 10^9^) (Figure 2). Their presence could be confirmed by Western blotting (Figure 1). ASAP1 regulates membrane traffic and cytoskeleton organization [19] and influences cell spreading and migration [20]. Directed by the Crk-like protein, it accumulates within the focal adhesion complex [21]. There, it co-localizes with PXN and VCL, which are able to bind to each other [19,22], and ASAP1 associates also with the focal adhesion kinase. Under unknown conditions, ASAP1 may cause a repositioning of PXN and focal adhesion kinase within the focal adhesion complex, which retards cell spreading [23]. ASAP1 has also been observed to bind to ARF6 [24]. This binding appears to facilitate the recruitment of ASAP1 to the focal adhesion complex [25]. Interestingly, the *ASAP1* gene was up-regulated in adherent FTC-133 cells after 24 h of incubation [11]. However, after 10 d in space, the *ASAP1* gene was 7-fold down-regulated in adherent cells, while it remained unregulated in spheroid cells [5].

The p130cas and MAPK8 (Mitogen-activated protein kinase 8) protein, which both are members of the integrin signaling pathway, were detected in sample 3 but not in sample 2. The p130cas protein (see BCAR1, breast cancer anti-estrogen resistance protein 1, in Figure 2) interacts also with PXN, VCL, and PTK2. Paxillin is a membrane protein that regulates cell-matrix interaction, and associates with p130cas [26]. When PXN-p130cas complexes are phosphorylated, they constitutively activate cell migration by inducing gene 5 proteins (RAC1) to abolish shear stress induced cell polarization [27]. Phosphorylation occurs by interaction with focal adhesion kinase after cell adhesion [28,29]. p130cas interacts also with VCL in focal adhesion complexes that mediate cell-matrix interactions in the presence of PXN [30,31]. MAPK8 proteins signal shear stress to focal adhesion sites in endothelial cells [32]. Its activation is controlled by ARF6 [33]. In human multiple myeloma cells, the expression of MAPK8 and interleukin-8 genes is suppressed simultaneously by azidothymidine [34]. Recently, we found that MCF-7 breast cancer cells enhance interleukin-8 gene expression only in adherent cells treated just as the cells of sample 3 [35]. In addition, when a population of FTC-133 was flown through the orbit on a Chinese rocket (Shenzhou-8 space mission), the *MAPK8* gene was up-regulated in the cells remaining adherent. This suggested that a strengthening of the *MAPK8* signal over extracellular shear forces could hinder the cells to aggregate to floating spheroids. The conclusion has still to be confirmed, as the c-Jun N-terminal kinase 1 (JNK-1), which is also called MAPK8, was, according to Figure 1A, similar in MCS and adherent cells, both harvested from culture condition 2 and 3.

Taken together, the results shown in Figure 2 suggest that composition and interaction of PXN, VCL, PTK2, and ARF6 are strongly influenced by ASAP1 when cells form spheroids (sample 2). When FTC-133 cells grow in monolayers, even under conditions of microgravity (sample 3), the same proteins are predominantly affected by p130cas and MAPK8. Enhanced ASAP1 appears to weaken the focal adhesion complexes, so that the removal of sedimentation forces together with minimal shear forces present in culture flasks on the RPM may trigger the cells to detach from the bottom of the culture flask but anchor to neighboring cells [6,36]. At the same time, a predominance of p130cas appears to strengthen lamellipodia [28] as well as cell-matrix interaction [30] via PXN [26], which facilitates continued adherent growth.

**Figure 3:**
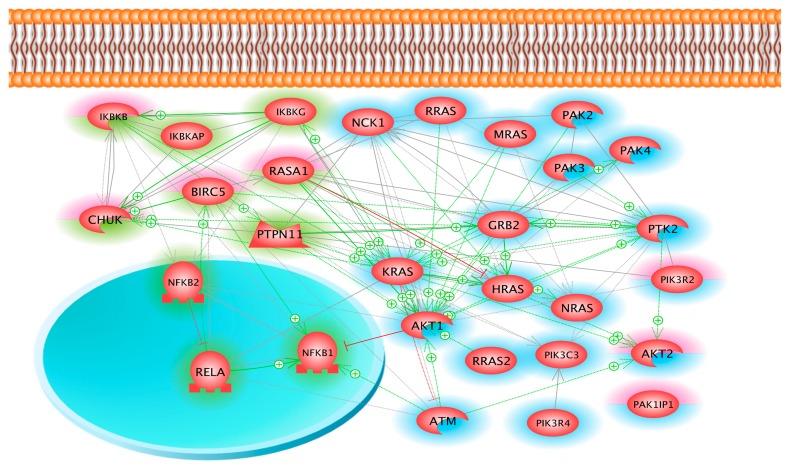
The interaction and localization of proteins, belonging to the angiopoietin signaling pathway and found in samples 2 and/or 3. All proteins marked by a uniform green or blue rim were found in samples 2 and 3, while the proteins of *CHUK*, *IKBKB*, *BIRC5*, *RAS1*, *PIK3R2*, *PAK1IP1* and *AKT2*, marked by a upper half red rim were found only in sample 3. Proteins of *CHUK*, *IKBKB*, *BIRC5*, *RAS1*, *IKBKAP*, *IKBKG*, *PTPN11*, *NFKB1*, *NFKB2* and *RELA* belong to the angiopoietin pathway only. The rest of the proteins belong to the integrin signaling and the angiopoietin pathway simultaneously. Arrows indicate interaction, while T-bars show inhibition; schemes of a membrane bilayer, a nucleus (circle) and a mitochondrion outline localization.

Furthermore, we looked at the angiopoietin pathway. It comprises 66 proteins, 22 of which were detected in sample 2. In sample 3, the 22 proteins of sample 2 were also found plus an additional seven (Figure 3). Nineteen of the 29 proteins detected in samples 2 and 3 belong to both the angiopoietin and the integrin signaling pathways. The finding of proteins of the angiopoietin pathway was not surprising; we had repeatedly found increased gene expression and protein secretion of vascular endothelial growth factor (VEGF) of the neutrophil gelatinase-associated lipocalin, osteopontin, and the interleukins 6 and 17 in thyroid cancer cells, suggesting that factors of the angiopoietin pathway may play a role in spheroid formation of FTC-133 cancer cells [5,8,37]. In this proteome study, no membrane-bound receptor for VEGF or any other of the factors mentioned above could be detected in the thyroid cells. Of the ten proteins belonging to the angiopoietin but not the integrin signaling pathway, four proteins were found in sample 3 (adherent cells) but not in sample 2 (spheroids). One of these was Ras GTPase-activating protein 1 (RASA1), which can bind to paxillin and to PTK2 and supports cell migration and surface ruffling [38,39]. In addition, it forms a complex with survivin [40], which accumulates CHUK protein (Inhibitor of nuclear factor kappa-B kinase subunit alpha) in the nucleus [41]. There, CHUK protein together with IkBKB (Inhibitor of nuclear factor kappa-B kinase subunit beta) regulates nuclear factor kappa B activity [11,42].

## 3. Discussion

The proteome analysis of FTC-133 thyroid cancer cells cultured under various conditions revealed more than 5900 proteins of this cell line. The proteins detected represent about 40% of proteins possibly produced in human cells [13]. Their quantities ranged from 10^7^ to 10^11^ LfQ values. Advanced analysis of the detected proteins in regard to their association to the integrin signaling pathway and the angiopoietin pathway challenged the following conclusions: In cells forming spheroids during three days of culture under s-µ*g*, the levels of CAV-1 and p130cas proteins are reduced, but ASAP1 production is enhanced. Under this condition, proteins PXN, VCL, and PTK2 may be positioned within the focal adhesion complex in a way that favors cell detachment from the bottom of a culture flask and mutual attachment. The continuation of adherent growth could be supported by accumulation of p130cas protein in individual cells. In order to prove this hypothesis, we shall investigate the structural changes of the cell adhesion complex applying methods described recently [43].

## 4. Materials and Methods

### 4.1. Cell Culture

FTC-133 human follicular thyroid carcinoma cells [44] were cultured in RPMI 1640 (Life Technologies, Naerum, Denmark) medium supplemented with 10% fetal calf serum (Biochrom AG, Berlin, Germany) and 1% penicillin/streptomycin (Life Technologies, Naerum, Denmark) under standard cell culture conditions at 37 °C and 5% CO_2_. Prior to culturing under different conditions, 1 × 10^6^ cells were counted and seeded into T25 cm^2^ vented cell culture flasks (Sarstedt, Newton, MA, USA). Twelve of these T25 cm^2^ culture flasks each containing 10^6^ cells were incubated at 37 °C for two days until the cells formed sub-confluent monolayers. Afterwards, three flasks were put nearby the RPM (culture condition 1), while another three were mounted on the RPM (culture conditions 2 and 3). Then, each of these flasks was incubated for another three days prior to harvest. The six remaining flasks continued to be cultured for another three days under normal gravity until the monolayers had reached confluence. Then, again, three of the six flasks were put nearby the RPM (culture condition 4), while the other three were mounted on the RPM (culture conditions 5 and 6). These flasks were also incubated for another three days until harvest (Table 1). Therefore, the main difference between culture conditions 1 and 4 as well as between culture conditions 2 and 3 and culture conditions 5 and 6 is the length of the period of pre-incubation. These cell culture samples were used for MS. For the Western blot analyses, we repeated these experiments (*n* = 5 per condition).

### 4.2. Random Positioning Machine

A desktop RPM (Airbus Defense and Space, Leiden, The Netherlands) was placed in a commercially available incubator at 37 °C and 5% CO_2_. The RPM was used in real random mode with random speed and random interval and a maximum speed of 75°/s. The flasks were fixed to the central frame, as near as possible to the center of rotation, and were rotated for 3 days. Corresponding static normal gravity controls (1*g*) prepared in parallel were stored next to the device in the same incubator during the time of rotation. Each flask was completely filled with complete medium, taking care that no air bubbles remained in the cell culture flasks in order to minimize shear stress. The mode of action and the effectiveness of this machine at preventing cell sedimentation have been described previously [45,46].

### 4.3. Cell Harvest

First, the supernatant of each T25 cm^2^ culture flask was collected and centrifuged at 4°C for spheroid collection. After centrifugation, the supernatant was carefully aspirated, and the spheroids were collected, washed in phosphate buffered saline (PBS, Gibco, Life Technologies, Naerum, Denmark), and stored in liquid nitrogen. To harvest the adherent cells, 5 mL of ice-cold PBS were carefully added to each T25 cm^2^ flask. The supernatant was then aspirated and the cells were scraped off with a scraper. The cell suspension was collected and centrifuged at 4 °C. The PBS was discarded and the dry pellet was washed with PBS and stored in liquid nitrogen.

### 4.4. Mass Spectometry

Cells were lysed in a buffer containing 6 M guanidium hydrochloride, 20 mM TCEP (tris(2-carboxyethyl)phosphine), and 40 mM chloroacetamide in 25 mM Tris pH 8.0. Lysis buffer, preheated to 95 °C, was added to the cells and sonicated using a Bioruptor plus water bath sonicator (Diagnode, Seraing, Belgium). The lysates were heated again at 95 °C for 2 min, followed by one more sonication step at maximum power settings for ten cycles. Following complete lysis, the sample was diluted 10-fold with 25 mM Tris pH 8.0 and digested overnight at 37 °C with endoproteinase Lys-C (Wako Chemicals GmbH, Neuss, Germany) at a 1:50 protein ratio. The digested peptides were then purified and concentrated on three plugged SDB-XC StageTip [47].

For the liquid chromatography-mass spectrometry analysis, about 2 µ*g* of peptides were loaded onto a 15 cm, 75 µm I.D column packed with 1.9 µm C18 beads (Maisch GmbH, Ammerbuch, Germany) using the Thermo easy n-LC 1000 system (Thermo Scientific, Waltham, MA, USA) and were separated over a 120-min gradient with buffer A (0.1% formic acid) and buffer B (0.1% formic acid and 80% acetonitrile). The LC (liquid chromatography) column was maintained at a constant temperature of 45 °C using a column oven (Sonation, Biberach, Germany). The peptides eluting from the column were directly sprayed into a Q Exactive HF mass spectrometer (Thermo Scientific, Waltham, MA, USA) via a nano-electrospray ionization source (Thermo Scientific, Waltham, MA, USA) [48]. The mass spectrometer was operated in a data-dependent top 15 mode. Survey scans and fragmentation scans were acquired at resolutions of 60,000 and 15,000 respectively (*m*/*z* = 200). Fragmentation was performed on precursors isolated within a window of 1.4 *m*/*z* with a normalized collision energy setting of 27.

Raw data from the mass spectrometer were processed using MaxQuant computational proteomics platform version 1.5.2.22 (Computational Systems Biochemistry, Max-Planck-Gesellschaft, Munich, Germany) [49] using the standard parameters. Relative protein concentration was performed using the LfQ algorithm (label-free quantitation) as described elsewhere [50].

### 4.5. Pathway Analysis

To investigate and visualize the original localization and the mutual interactions of detected proteins, we entered relevant UniProtKB entry numbers in an Elsevier Pathway Studio® v.11 software (Elsevier Research Solutions, Amsterdam, The Netherlands). To assign detected proteins to canonical pathways, the Ingenuity Pathway Analysis (IPA) with Advanced Analytics client (CL) (Qiagen GmbH, Hilden, Germany) was applied, also entering relevant UniProtKB entry numbers.

### 4.6. Western Blot

Western blotting, immunoblotting, and densitometry were performed as described earlier [17]. We used the biorad ChemiDoc XRT+ device. The antibodies used to detect and quantify the antigens are listed in Table 3. The applied secondary antibody, a Horseradish peroxidase (HRP)-linked antibody was utilized at a dilution of 1:4000 (Cell Signaling Technology, Inc., Danvers, MA, USA). In addition, we used glyceraldehyde 3-phosphate dehydrogenase (GAPDH; dilution: 1:1000). Ponceau S red staining was used as an alternative to housekeeping proteins as loading controls. The membranes were analyzed using ImageJ software (U.S. National Institutes of Health, Bethesda, MD, USA; http://rsb.info.nih.gov/ij/), for densitrometric quantification of the bands. Ponceau S was evaluated according to [51]. Statistical analyses were performed as previously published [17].

## Figures and Tables

**Table 1:** Cell preparation and number of proteins detected.

Culture Condition	1	2 and 3		4	5 and 6
Sample Number	#1	#2	#3	#4	#5
Pre-incubation	2 days	2 days		5 days	5 days
Following 3 days incubation under…	1*g*	s-µ*g*		1*g*	s-µ*g*
Kind of growth at time of harvest	Adherent	Spheroid	Adherent	Adherent	Adherent
Number of proteins detected	4419	4505	4544	4621	4961

**Table 2:** Percentage of proteins of cellular compartments covered by the detected proteins.

Cellular Compartment	Sample 1	Sample 2	Sample 3	Sample 4	Sample 5
Secreted proteins	19%	19%	19%	20%	21%
Membrane	27%	27%	27%	28%	29%
Nucleus	29%	29%	29%	30%	31%
Cytoplasm	29%	30%	30%	30%	32%
Golgi apparatus	31%	31%	32%	34%	35%
Cytoskeleton	33%	33%	34%	35%	37%
Endoplasmic reticulum membrane	35%	36%	37%	36%	39%
Endoplasmic reticulum	36%	36%	37%	36%	39%
Perinuclear region of cytoplasm	37%	38%	37%	38%	40%
Intracellular-membrane-bounded organelle	37%	37%	38%	39%	40%
Endosome	38%	38%	38%	40%	41%
Cytosol	38%	39%	39%	40%	42%
Extracellular exosome	41%	43%	42%	44%	45%
Nucleoplasm	42%	42%	42%	42%	45%
Mitochondrion	49%	48%	49%	48%	50%
Nucleolus	50%	50%	50%	49%	50%
Nuclear speck	51%	50%	50%	50%	57%
Mitochondrial inner membrane	60%	60%	61%	61%	62%
Focal adhesion	63%	64%	65%	65%	68%
Mitochondrial matrix	65%	62%	63%	65%	63%
Spliceosomal complex	68%	69%	66%	66%	71%
Ribonucleoprotein complex	72%	72%	71%	72%	73%
Mitochondrial small ribosomal subunit	93%	93%	93%	93%	93%
Large ribosomal subunit	100%	100%	100%	100%	100%

**Table 3:** Antibodies applied for Western blot analysis*.*

Antibody	Dilution	Company	Molecular Weight	Catalog Number
Anti-JNK1	1/1000	Abcam	48 kDA	ab110724
Anti-FAK	1/1000	Abcam	125 KDA (119 kDA)	ab40794
Anti-Vinculin	1/1000	Abcam	130 kDA	ab18058
Anti-Caveolin-1	1/1000	Abcam	22 kDA	ab2910
Anti-NfκB p65	1/1000	Cell-Signaling	65 kDA	#C22B4
Anti-GAPDH	1/1000	Cell-Signaling	37 kDA	#5174

## Abbreviations

AD	Adherent cells
AKT1	RAC-alpha serine/threonine-protein kinase 1
ARF6	ADP-ribosylation factor 6
ASAP1	Arf-GAP with SH3 domain, ANK repeat and PH domain-containing protein 1
BIRC5	Baculoviral inhibitor of apoptosis repeat-containing 5; Survivin
CAV-1	Caveolin 1
CHUK	Conserved helix-loop-helix ubiquitous kinase; Inhibitor of nuclear factor kappa-B kinase subunit alpha (IKK-α)
GAPDH	Glycerinaldehyd-3-phosphat-Dehydrogenase
HRP	Horseradish peroxidase
IKBKB	Inhibitor of nuclear factor kappa-B kinase subunit beta
JNK1	c-Jun N-terminal kinase 1 or Mitogen-activated protein kinase 8
LfQ	Label-free Quantitation
MAPK8	Mitogen-activated protein kinase 8
MCS	Multicellular tumor spheroids
MS	Mass Spectrometry
NFκB	Nuclear factor kappa B
PAK1IP1	P21-activated protein kinase-interacting protein 1
PIK3R2	Phosphoinositide-3-kinase regulatory subunit 2
PTK2	Protein tyrosine kinase 2, focal adhesion kinase 1 (FAK1)
PXN	Paxillin
Ras1	Ras-like protein 1
r-µ*g*	Real microgravity
RPM	Random positioning machine
s-µ*g*	Simulated microgravity
VCL	Vinculin
VEGF	Vascular endothelial growth factor
